# Microcontroller-Assisted Compensation of Adenosine Triphosphate Levels: Instrument and Method Development

**DOI:** 10.1038/srep08135

**Published:** 2015-01-30

**Authors:** Jie-Bi Hu, Ting-Ru Chen, Yu-Chie Chen, Pawel L. Urban

**Affiliations:** 1 Department of Applied Chemistry, National Chiao Tung University, 1001 University Rd, Hsinchu, 300, Taiwan; 2 Institute of Molecular Science, National Chiao Tung University, 1001 University Rd, Hsinchu, 300, Taiwan

## Abstract

In order to ascertain optimum conditions for biocatalytic processes carried out *in vitro*, we have designed a bio-opto-electronic system which ensures real-time compensation for depletion of adenosine triphosphate (ATP) in reactions involving transfer of phosphate groups. The system covers ATP concentration range of 2–48 μM. The report demonstrates feasibility of the device operation using apyrase as the ATP-depleting enzyme.

Synthetic biology is an emerging field which aims to engineer new organisms, and create artificial biosystems without using native intact cells^1^,^2^,^3^,^4^. By taking advantage of biomolecules and biochemical reactions, it may – in a longer term – help to improve the quality of people's life^5^. Enzyme biocatalysis plays the key role in transformations of biomolecules in artificial systems. Some of the approaches take advantage of multi-enzyme reaction chains and networks. Many enzymes use adenosine triphosphate (ATP) as the source of chemical energy and phosphate groups. For example, Bujara *et al.*^6^ demonstrated a multi-enzyme system replicating glycolysis *in vitro*, which could serve for optimisation of complex multi-step biosyntheses. In cells, the levels of ATP are stabilised by the intrinsic homeostatic mechanisms. The energy charge of a cell describes the relative content of ATP with respect to other adenylate forms. It has been documented that the energy charge of living cells is stabilised to a great extent^7^. However, when reproducing biosynthetic processes *in vitro*, another way of stabilising ATP concentration is required. One study found that the productivity of *in-vitro* syntheses (*e.g.* cell-free protein synthesis) was limited by deficiency of ATP, owing to the depletion of this energy source^8^. In view of future applications of biocatalytic systems involving ATP-consuming enzymes, it is desirable to develop methods which could efficiently stabilise or regenerate ATP in cell-free syntheses.

Here we propose a device and method for the compensation of ATP levels in an *in-vitro* experiment, in which ATP is constantly depleted due to the presence of an ATP-hydrolysing enzyme. The approach involves combination of a biochemical reaction with real-time monitoring of the relative ATP content and automatic compensation – enabled by an electronic control unit. It takes advantage of an open-source software development kit (mbed), a compatible universal microcontroller, and a simple feedback control algorithm. In fact, universal electronic modules provide many opportunities for the development of low-cost and versatile analytical platforms^9^,^10^,^11^,^12^,^13^,^14^.

In the proposed compensation system, the mbed microcontroller (Fig. 1), programmed in C++ (Fig. 2), receives chemiluminescence data from the photoresistor circuit (Fig. S1), and it takes over the operation of syringe pump. If the chemiluminescence signal is too low, it triggers the syringe pump to supplement the reaction mixture with ATP until the signal is restored to the desired level. The injection of ATP is carried out in discrete steps which are executed with the maximum frequency of ~0.1 Hz. In addition, the chemiluminescence data are displayed on a miniature LCD screen and stored in the flash memory of the microcontroller for post-reaction evaluations of the amount of ATP used, and drawing conclusions on biochemical inertia of the studied biocatalytic system.

In order to characterise the home-made detection system, we injected aliquots of ATP solution with different concentrations. Subsequently, a calibration curve was created showing good correlation between the chemiluminescence signal and the ATP concentration within the range 2–48 μM (Fig. S2). The limit of detection is estimated to be ~1.1 μM ATP (based on the 3 × RMS noise criterion), which is satisfactory considering that an inexpensive low-performance light sensor was implemented. Using real-time mass spectrometry, we also confirmed the correlation of relative ATP depletion and AMP production in the course of luciferin/luciferase reaction conducted according to the described protocol (*cf.* Supporting Information; Fig. S3).

To demonstrate the feasibility of the compensation approach, a pair of experiments was conducted: In one variant, 16 nmol (16 μL, 1 mM) ATP was injected only once, *i.e.* 120 s after the start of monitoring. The light intensity immediately increased, and then gradually decreased over several minutes (Fig. 3A). In the other variant, the initial injection of ATP (16 nmol; 16 μL, 1 mM) was followed with multiple injections of small aliquots of the same reagent (8 nmol; 8 μL, 1 mM). The initial injection of ATP set the concentration of ATP in the reaction cell to be ~32 μM, while every additional injection contributed to ~16 μM increase of ATP. The program (loaded to the microcontroller) was set to inject ATP when the chemiluminescence intensity fell below 80% of the initial chemiluminescence – recorded right after injecting the first aliquot of the ATP solution. This resulted in a series of ATP injections which stabilised the level of chemiluminescence produced in the reaction cell (Fig. 3B). Although the exact timing of ATP additions – decided by the algorithm in real time – varies from run to run; in general, the system coped with the stabilisation of ATP levels over a 10-min period, as determined based on a series of replicate experiments (Fig. S4). A possible explanation of the differences in injection times is the stochasticity of mixing ATP solution with the reaction mixture. The amplitude differences (after the first injection) are attributed to the limited efficiency of turbulent mixing due to the fact that the reaction volume is small (0.5 mL).

Overall, the experimental data (Fig. 3B) show that the system is capable of maintaining ATP concentration away from the reaction equilibrium. It is interesting to note that – when the compensation device is switched off – the chemiluminescence signal disappears according to a non-linear characteristics (Fig. 3A): initially, the signal decreases rapidly, then it stabilises. The data points recorded during a 50-s period starting from the time point when chemiluminescence maximum was observed were fitted with a linear function (Fig. S5A), and the resulting slope values were used as a proxy for the initial reaction rate. By correlating these values with the initial ATP concentration in the reaction vial (Fig. S5B), the apparent Michaelis-Menten constant (*K*_M_) could be estimated to be ~22 μM ATP. Note well: this is a rough estimation considering that the method is not purposely optimised for *K*_M_ determinations, and the mean squared error of the fitted model in Fig. 5B is high. However, the estimated *K*_M_ value is in the order of the corresponding *K*_M_ values observed for the same enzyme (*cf.* Brenda database). Most importantly, it is pleasing to note that the proposed bio-opto-electronic compensation system is capable to maintain the ATP level higher than the *K*_M_ value of luciferase, at which the depletion of ATP is relatively fast. Based on this result, one can hypothesise that when the reaction mixture also contains other ATP-consuming enzymes (with high turnover rates), the system will be able to maintain ATP at the desired level.

Apyrase hydrolyses ATP to adenosine diphosphate (ADP) and adenosine monophosphate (AMP; *cf.* Fig. 1); therefore, it can be used to emulate the activity of ATP-consuming enzymes. To further prove the effectiveness of the proposed bio-opto-electronic system in the compensation of ATP depleted in the course of biocatalytic processes, different amounts of apyrase (200, 500 and 1000 μU) were spiked to the reaction mixture before the reaction was triggered by injecting the first aliquot of ATP (Figs. 3C–3H). The results obtained from the control experiments (compensation disabled) show the ATP decay rate is related to the amount of apyrase (Figs. 3C, 3E and 3G). High amount of apyrase gave rise to fast ATP decay. In fact, the rate of chemiluminescence decrease seems to be more dependable than recording the amplitude of the first peak, which might be affected by the low mixing efficiency and the related stochasticity. Importantly, the compensation system maintained the ATP level in the presence of apyrase representing an ATP-consuming enzyme (Figs. 3D, 3F and 3H). Moreover, the number of compensatory injections was related to the activity of apyrase. Interestingly, the frequency of compensatory injections was higher in the last few minutes of the experiment. When a large amount of apyrase was present in the reaction mixture, the system could no longer sustain the stable level of ATP (represented by chemiluminescence; *cf.* Fig. 3H) at the preset maximum frequency of injections (~0.1 Hz). The above effects can be due to various reasons: (i) inhibition of luciferase by the reaction products; (ii) significant depletion of reaction substrates other than ATP (luciferin, oxygen); and (iii) dilution of the reaction mixture by the added ATP solution. For example, the intermediates of the luciferase-catalysed reaction (luciferyl-AMP and dehydroluciferyl-AMP) as well as the end-products (oxyluciferin, AMP and pyrophosphate) have been shown to inhibit luciferase^15^,^16^,^17^,^18^. Therefore, future work should combine the proposed bio-opto-electronic compensation system with a biochemical compensation strategy for recycling or removing the reaction end-products. However, the result obtained from a control experiment (Fig. S6) suggests that inhibition of luciferase by AMP is not significant under the conditions used here. One shall also note that while increasing the injection frequency limit (in the program loaded to the microcontroller) would allow the system to inject larger volumes of ATP solution, it would also lead to the further dilution of the reactants causing failure of compensation. This problem can be rectified by increasing the concentration of ATP in the solution injected by the syringe pump. It is also necessary to consider the availability of other substrates of the reaction. The required amount of luciferin can be estimated taking into account the amount of the injected ATP. For example, in Fig. 3A, the total amount of the ATP involved in the reaction is 16 nmol, while in Fig. 3B (compensation on), the total amount of the ATP involved in the reaction is 64 nmol. This is smaller than the amount of luciferin (199 nmol) – initially present in the reaction cell. Hence, the reaction is limited by ATP, not luciferin. When an ATP-decomposing enzyme (apyrase) is present in the sample, the total amount of ATP dispensed by the compensation system is greater – for example, 168 nmol in Fig. 3H. However, in this case, a large portion of ATP is being decomposed by apyrase, not luciferase, which decreases the chance for the luciferin/luciferase reaction being limited by luciferin content. Nevertheless, in the case of long-term experiments, luciferin may need to be supplied in larger quantities, or even added to the reaction mixture along with ATP during the subsequent compensatory injections, to avoid slowing down the reaction due to the decreasing concentration of this auxiliary substrate.

While in the above tests of the compensation system, low concentrations of ATP – which are within the linear range of the method (*cf.* Fig. S2) – were used, the system can be operated at higher concentrations of ATP (outside the linear range) – provided that the present ATPase can rapidly bring the ATP concentration down (*cf.* Fig. S7). Nevertheless, stabilisation of ATP at higher concentrations (>48 μM) may not be as efficient and accurate as in the case of lower concentrations (within the linear range). In order to extend the dynamic range of the method, one may consider fitting the reaction chamber with a sampling device that would collect aliquots of the reaction mixture and conduct on-line dilution prior to incubation with the luciferin/lucifease assay solution and detection. This would allow performing the measurements at higher concentrations of ATP making the approach more compatible with molecular biology experiments. One can also improve the detection system by replacing photoresistor with photodiode. Further tests of the current system also showed the possibility of implementing the compensation procedure while operating with more complex samples (breast Hs 578T cell extract; Fig. S8), which gives the promise for future practical applications of the proposed method. However, the anticipated usefulness of the method is yet to be verified when it is applied to real-world biosynthetic reactions, for example – cell-free translation.

In conclusion, we have proposed a simple bio-opto-electronic system capable of maintaining the ATP level far from equilibrium in spite of the presence of ATP-hydrolysing enzymes (luciferase, apyrase) in the reaction mixture. The system takes advantage of inexpensive and widely available components, and relies on the use of an inexpensive universal microcontroller and simple programming. It should be noted that ATP can also be regenerated enzymatically with pyruvate kinase using ADP and phosphoenolpyruvate as substrates^19^. On the other hand, regeneration of ATP from AMP would normally require the use of two enzymes. The proposed microcontroller-assisted compensation method is complementary to those enzymatic regeneration systems. Its advantage is that it only requires the use of one enzyme (luciferase) and inexpensive electronics. Its disadvantage is that it cannot remove the by-products of reactions, which may inhibit the reactions in longer reaction runs and (in the current version) has a limited dynamic range. While this report shows a technological improvement related to conducting biocatalytic reactions *in vitro*, further work is necessary to demonstrate its usefulness in the synthesis of new compounds.

## Author Contributions

J.-B.H. wrote the program, conducted experiments, treated data, and prepared figures. T.-R.C. assisted at some experiments. P.L.U. provided the research idea, designed the electronic system, wrote the program, and discussed the results. Y.-C.C. discussed the results. All authors reviewed the manuscript.

## Supplementary Material

Supplementary Information Supplementary Information

## Figures and Tables

**Figure 1:**
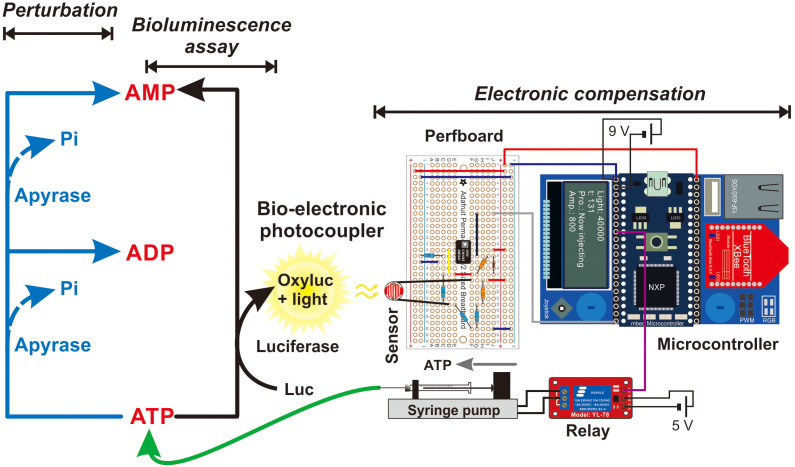
Simplified schematic of the bio-opto-electronic system for the compensation for ATP depletion comprising reaction chamber, photosensor, mbed microcontroller, relay and syringe pump. Apyrase was used as a model ATP-consuming enzyme. For a detailed electronic diagram, see Fig. S1.

**Figure 2:**
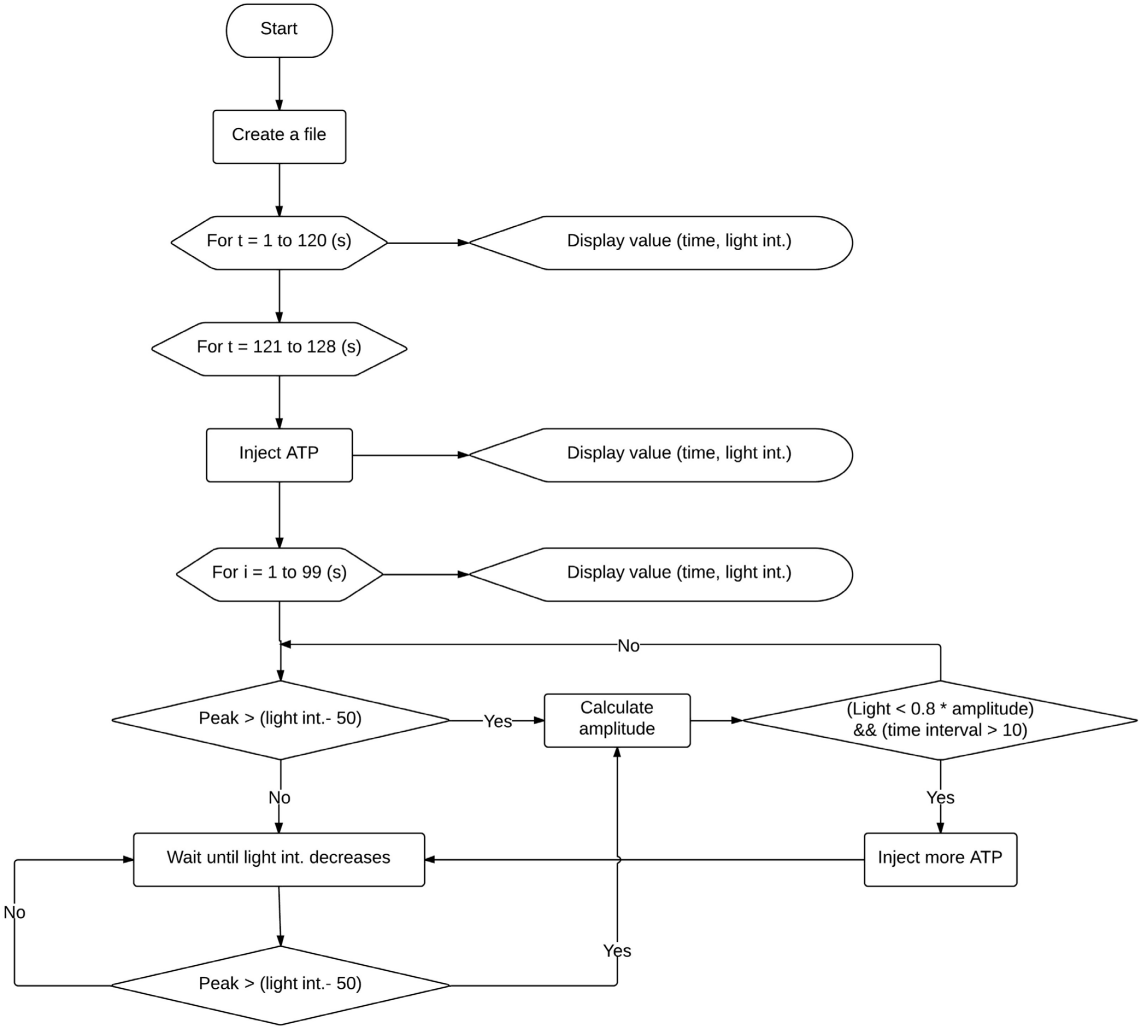
Block diagram of the algorithm loaded to the mbed microcontroller.

**Figure 3:**
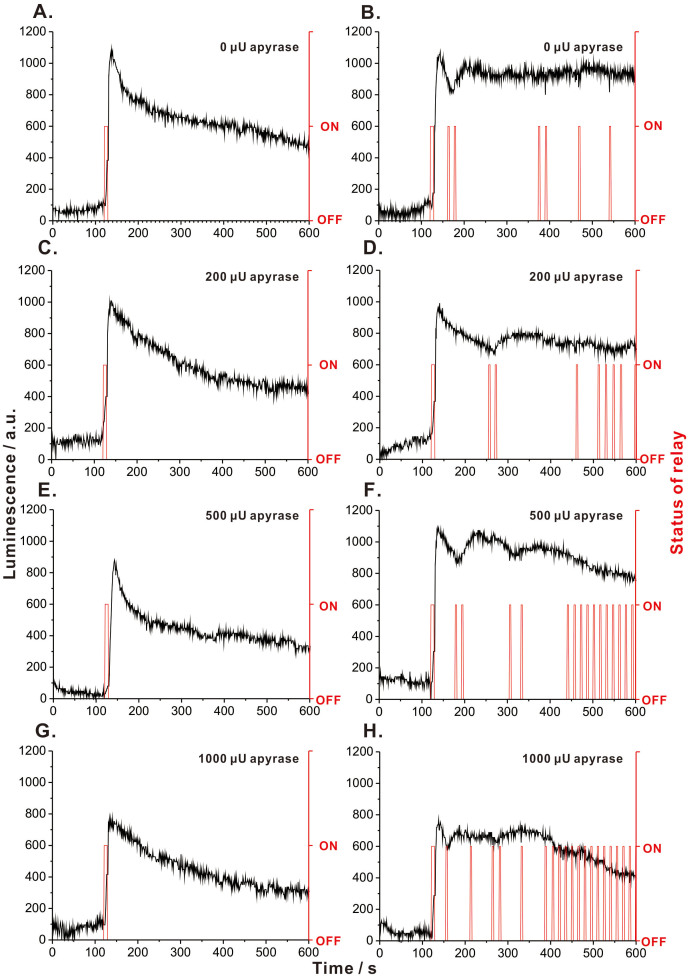
Experimental data proving the effectiveness of the bio-opto-electronic system for the compensation for ATP depletion. (A,C,E,G) Control: aliquot of ATP injected at the beginning of experiment; no compensation. (B,D,F,H) Aliquot of ATP injected at the beginning of experiment; compensation enabled. (A,B) Luciferase reaction alone (no apyrase present). (C-H) Apyrase added to the reaction mixture to emulate the presence of an ATP-consuming enzyme during biosynthesis. Different amounts of apyrase tested (200–1000 μU). For replicates of the result in (B), see Fig. S4.
