# Supplementary material for: Microcontroller-Assisted Compensation of Adenosine Triphosphate Levels: Instrument and Method Development
Source: Sci Rep. 2015 Jan 30;5:8135. doi: 10.1038/srep08135 (PMC4311256; doi:10.1038/srep08135)
Supplement: Supplementary Information [file srep08135-s1.doc]

**SUPPORTING INFORMATION**

**Microcontroller-Assisted Compensation of Adenosine Triphosphate Levels: Instrument and Method Development**

Jie-Bi Hu,^1^ Ting-Ru Chen,^1^ Yu-Chie Chen,^1,2^ and Pawel L. Urban^1,2^,*

^1^ Department of Applied Chemistry, National Chiao Tung University

1001 University Rd, Hsinchu, 300, Taiwan

^2^ Institute of Molecular Science, National Chiao Tung University

1001 University Rd, Hsinchu, 300, Taiwan

* Corresponding author: Prof. P.L. Urban

Fax +886-3-5723764

E-mail: plurban@nctu.edu.tw

**Experimental section**

***Materials***

Adenosine triphosphate (ATP), ethylene diamine tetraacetic acid (EDTA), D-luciferin, and magnesium sulfate were purchased from Sigma-Aldrich (St Louis, MO, USA). Ammonium acetate was purchased from Fluka (Zwijndrecht, Netherlands). LC-grade water was purchased from Merck (Darmstadt, Frankfurt, Germany). Apyrase was purchased from New England Biolabs (Ipswich, MA, USA). Luciferase (E.C. 1.13.12.7) from North American firefly (*Photinus pyalis*) was purchased from Promega (Madison, WI, USA).

***Enzymatic reaction system***

The enzymatic reaction system contains enzymes, substrates, buffer and other essential reaction components. The composition of the reaction medium present in the reaction cell was the following: water, 20 mM ammonium acetate, 0.2 mM magnesium sulfate, 0.2 mM EDTA, 0.4 mM D-luciferin and 0.1 mg mL^-1^ luciferase (~ 2.5 × 10^11^ U mL^-1^). After the microcontroller recorded blank signal for 120 s, 16 nmol ATP was injected to the cell to trigger the reaction (unless otherwise noted). The total reaction volume was set at 500 μL (including the first aliquot of ATP injected to initiate the reaction).

Firefly luciferase catalyses oxidation of D-luciferin in the presence of oxygen, magnesium cation, and ATP (DeLuca, M. & McElroy, D. W. Kinetics of the firefly luciferase catalyzed reactions. *Biochemistry* **13**, 921-925 (1974); Dukhovich, A., Sillero, A. & Sillero, M. A. G. Time course of luciferyl adenylate synthesis in the firefly luciferase reaction. *FEBS Lett.* **395**, 188-190 (1996); Viviani, V. R. The origin, diversity, and structure function relationships of insect luciferases. *Cell Mol. Life Sci.* **59**, 1833-1850 (2002)), according to the equations:

$\text{luciferase}+\text{luciferin}+\text{ATP}\to(\text{luciferase}-\text{luciferyl AMP)}+\text{PPi}$ (eq. 1)

$\text{(luciferase}-\text{luciferyl AMP)}+\text{O}_{2}\to\text{luciferase}+\text{oxyluciferase}+\text{AMP}+\text{CO}_{2}+h\nu$ (eq. 2)

ATP reacts with luciferase, magnesium ion and D-luciferin to form a complex (luciferase-luciferyl-adenylate) and pyrophosphate. This complex then reacts with oxygen to emit light. The process of chemiluminescence does not require any external source of light, and provides the means to detect and quantify ATP with high sensitivity. Therefore, luciferase has become widely used in chemical biology and drug discovery-related studies (Thorne, N., Auld, D. S. & Inglese, J. Apparent activity in high-throughput screening: origins of compound-dependent assay interference. *Curr. Opin. Chem. Biol.* **14**, 315-324 (2010); Thorne, N., Inglese, J. & Auld, D. S. Illuminating insights into firefly luciferase and other bioluminescent reporters used in chemical biology. *Chem. Biol.* **17**, 646-657 (2010)).

***Bio-opto-electronic compensation system***

The mbed LPC1768 microcontroller (NXP Semiconductors, Eindhoven, Netherlands), equipped with a high performance ARM Cortex-M3 Core, was used along the mbed application board (NXP Semiconductors). The analogue input of the microcontroller was connected to an operational amplifier circuit fitted with photoresistor (8-20 kΩ) as the sensor of luminescence (**Fig. 1** and **S1**). To program the microcontroller, we used the on-line C++ compiler (mbed website, https://mbed.org/accounts/login/?next=%2Fcompiler%2F (viewed on 19/06/2014)). The algorithm (**Fig. 2**) collects data from the analogue input saving them into an ASCII file. It also decides when aliquots of ATP solution should be added to the reaction mixture. This is done by triggering the picoliter volume syringe pump KDS Legato 130 (Holliston, MA, USA) interfaced to the mbed via a relay module YL-78 (Centenary Materials, Shanghai, China). When the algorithm decides to add more ATP to reaction cell, the syringe pump injects ATP solution (8 nmol; 8 μL, 1 mM) to the reaction cell (unless otherwise noted).

In order to prevent the influence of ambient light on the readout, the reaction chamber was made of a cardboard sheet lined with aluminium foil. It housed a 2-mL glass reaction cell (2-SVW; Thermo Fisher Scientific, Langerwehe, Germany) placed inside a 10-mL amber-glass vial (SV-10; Nichiden-Rika Glass, Kobe, Japan). The 10-mL glass vial was additionally lined with aluminium foil inside and outside. In order to improve stability and reproducibility, the reaction chamber was placed inside a small thermoshaker (Vortemp 56 EVC; National Labnet Company, Woodridge, NJ, USA) set to 200 rpm and 30 °C. In the additional experiments (**Figs. S6-S8**), we used a larger thermoshaker (LM-420D; Yihder, Taipei, Taiwan).

***Preparation of cell extract***

A 1-mL aliquot of fresh Hs 578T cell suspension (10^6^ cells mL^-1^ in Dulbecco’s Modified Eagle Medium) was transferred into a 2-mL microcentrifuge tube, and centrifuged (rotor diameter: ~ 5.9 cm; miniSpin plus; Eppendorf, Hamburg, Germany) at 2000 rpm for 5 min. The supernatant was discarded, and the cells were resuspended in 1 mL 20 mM ammonium acetate and centrifuged at 2000 rpm for 5 min in order to remove residual medium. This washing step was conducted 3 times. Eventually, after the supernatant was discarded, the pellet was resuspended in 1 mL 20 mM ammonium acetate. The cell suspension was placed in an ultrasonic bath (600 W; DC600H; Delta, Taipei, Taiwan) for 1 h. The lysate was centrifuged at 7000 rpm for 5 min. The supernatant was stored at 4 °C till the experiment (no longer than 24 h).

***Real-time monitoring of luciferin/luciferase reaction by mass spectrometry***

In order to perform real-time monitoring of luciferin/luciferase reaction by mass spectrometry, a 484-μL aliquot of the reaction mixture was loaded to a 2-mL microcentrifuge placed in a small thermoshaker (ThermoMixer C; Eppendorf, Hamburg, Germany) set to 300 rpm and 30 °C. Herein, we implemented an ambient ionisation technique called Venturi easy ambient spray-ionisation (V-EASI; Santos, V. G. *et al.* Venturi easy ambient sonic-spray ionization. *Anal. Chem.* **83**, 1375-1380 (2011)). The Venturi pump (operated with nitrogen pressurised to 1.38 × 10^5^ Pa) aspirated the reaction solution from the microcentrifuge tube, and sprayed it in front of the orifice of the ion-trap mass spectrometer (amaZon speed; Bruker Daltonics, Bremen, Germany). A 39-cm fused silica capillary (ID 150 μm, OD 375 μm; GL Science, Tokyo, Japan) was used to transfer the sample continuously from the reaction chamber towards the ion source. The mass spectrometer was operated in the alternating polarity mode. The voltage applied to the ion transfer capillary was oscillating between -4500 V and 4500 V. The end-plate offset was set to 500 V. The flow rate of dry gas (nitrogen) was set to 5 L min^-1^, and the temperature of the heater was set to 250 °C. The mass range was set to 300 to 800 u e^-1^. Eventually, negative-ion spectra were used to obtain extracted-ion currents corresponding to [AMP-H]^-^ (346 u e^-1^), [ADP-H]^-^ (426 u e^-1^), and [ATP-H]^-^ (506 u e^-1^). Please note that a small signal of the [ADP-H]^-^ ion was recorded due to in-source decay.

**Additional figures**


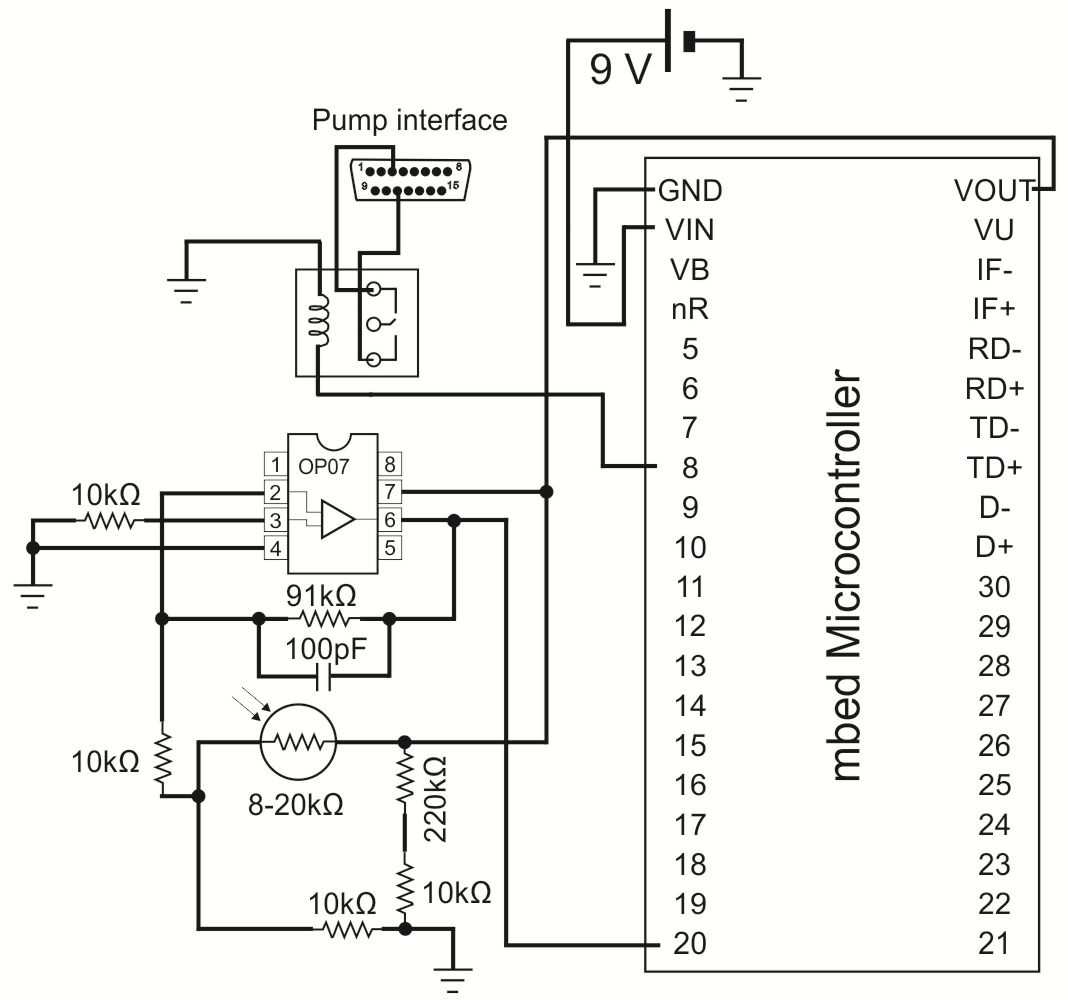


**Figure S1.** Electronic circuit used to record chemiluminescence data and to compensate for the depletion of ATP by triggering the syringe pump (*cf.* **Fig. 1**).


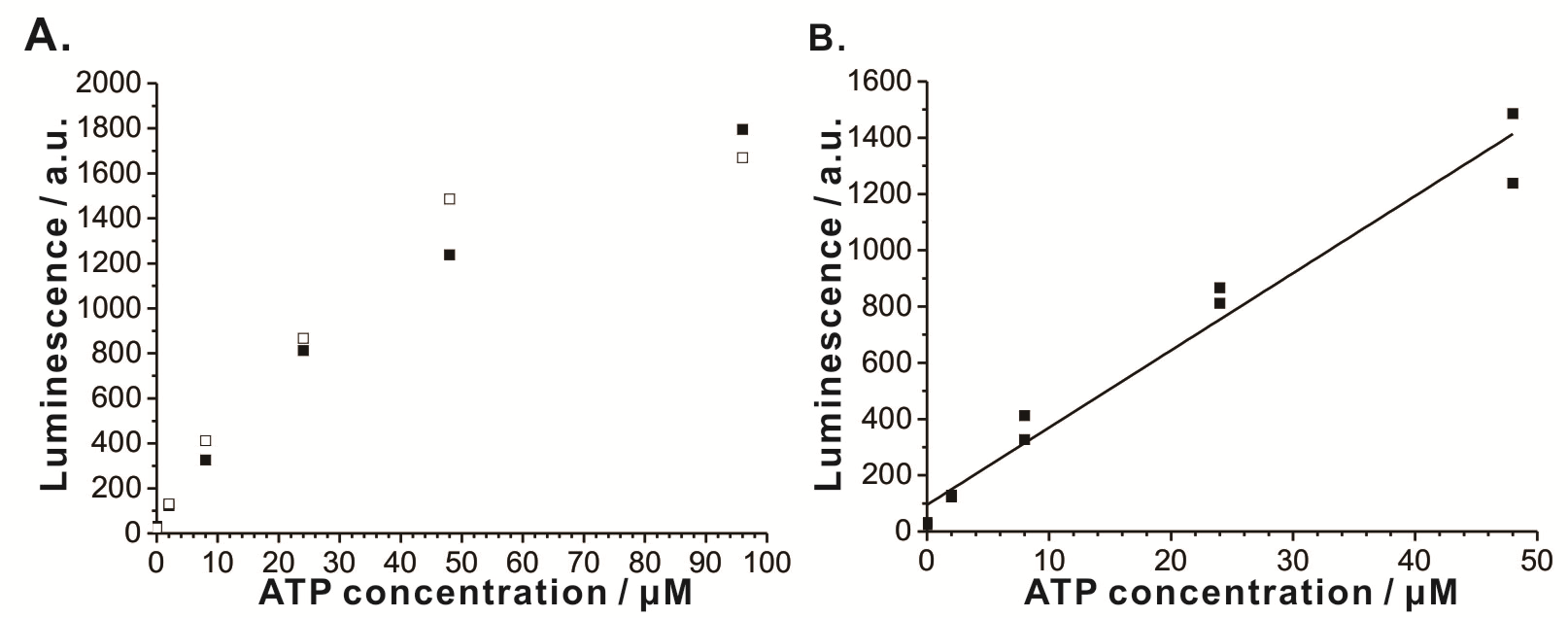


**Figure S2.** Calibration plot for the measurement of ATP using the proposed system (*cf.* **Fig. 1**). No apyrase was added. (A) Full-range dependence of chemiluminescence on ATP concentration. (B) Linear function (*Chemiluminescence* = (27.5±1.8) *C* + (94.6±40.6), fitted to the narrow range (0 – 48 μM) of the chemiluminescence/ATP characteristics (*R*^2^ = 0.9675).


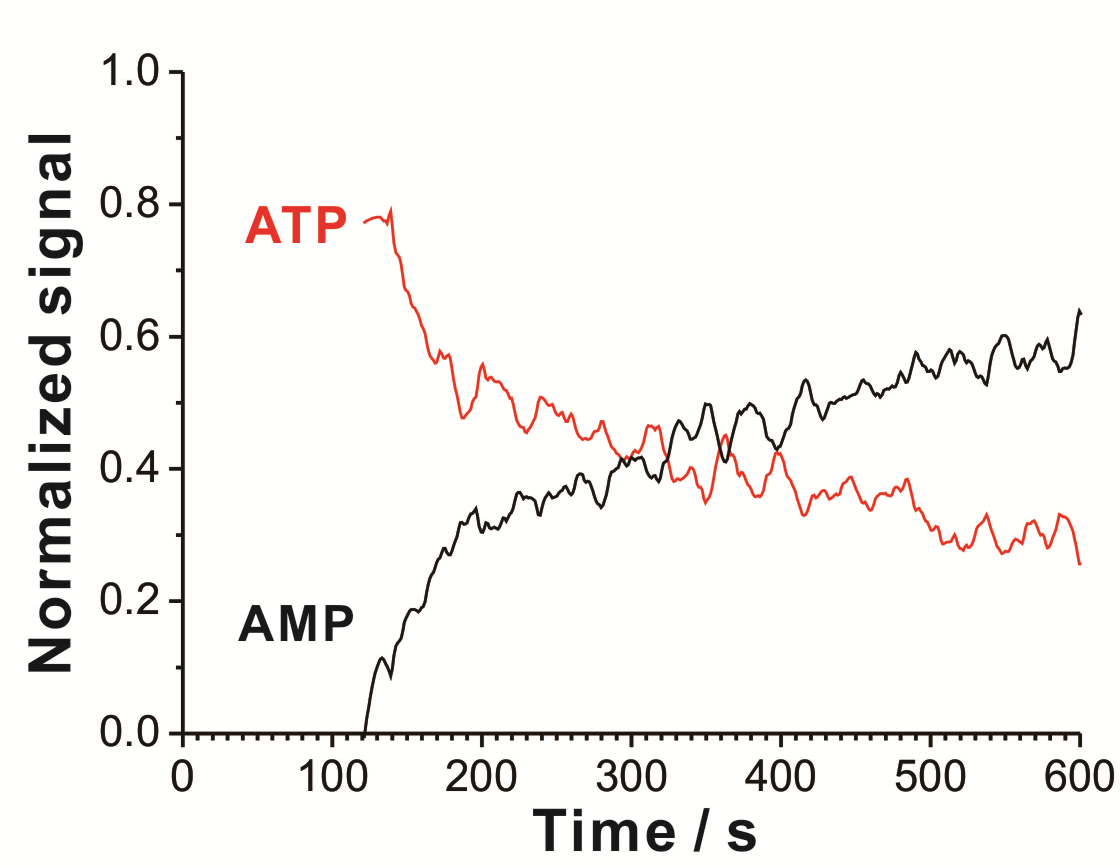


**Figure S3.** Real**-**time monitoring of luciferin/luciferase reaction by Venturi easy ambient sonic-spray ionisation (V-EASI) mass spectrometry. An aliquot of ATP (16 μL 1 mM stock solution) was injected at the beginning of the experiment (120 s after the start of data acquisition) to the final concentration 32 μM. The normalised signals were computed based on the extracted ion currents according to the following formulae: ATP/(AMP+ADP+ATP) for ATP (red line) and AMP/(AMP+ADP+ATP) for AMP (black line). Savitzky-Golay smoothing was applied to decrease the noise. In this experiment, ATP compensation was disabled.


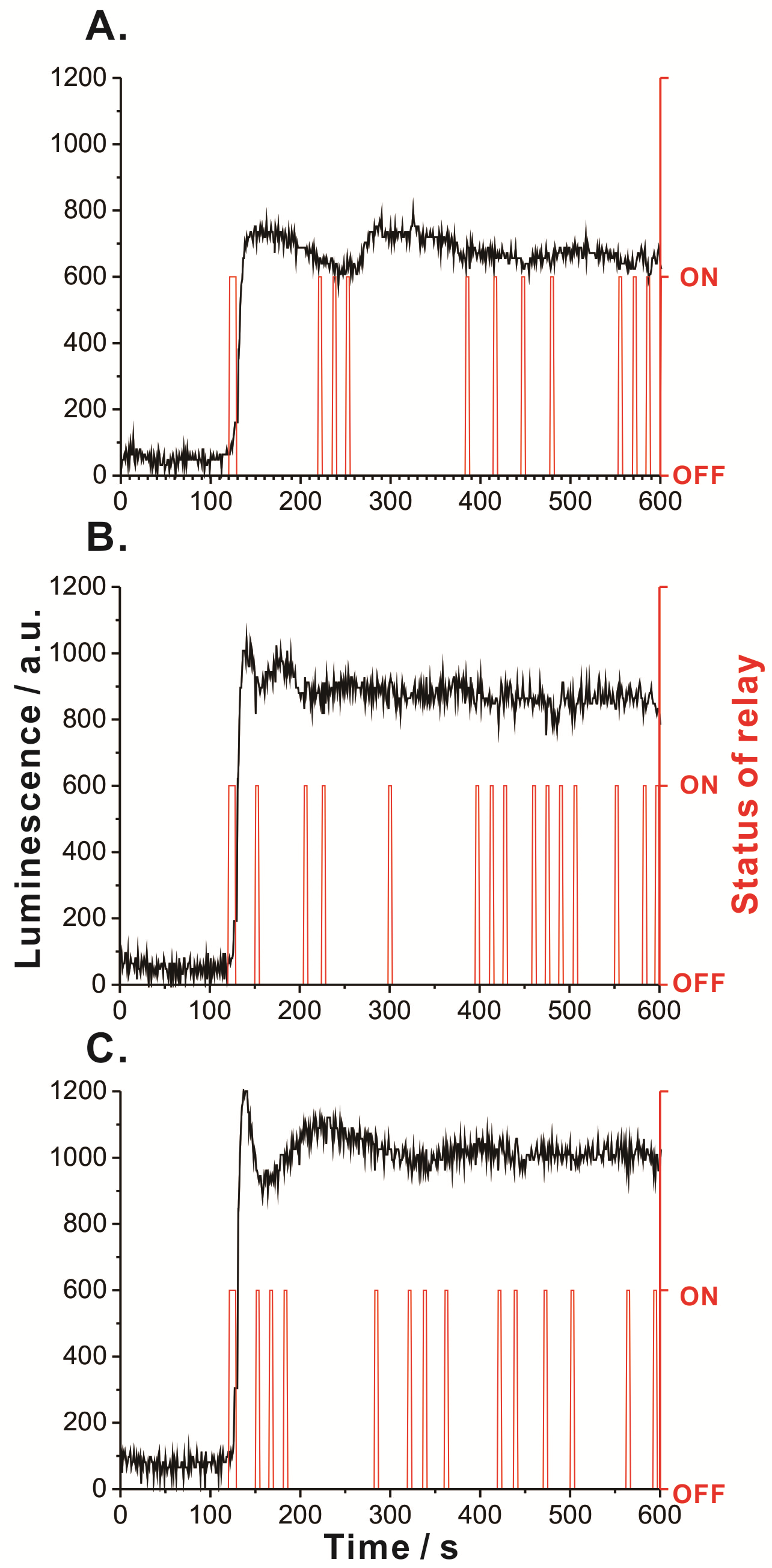


**Figure S4.** Bio-opto-electronic compensation of the depletion of ATP using the proposed system (*cf.* **Fig. 1**). Three replicate results. Experimental conditions are the same as in **Fig. 3B**. Relative standard deviation of signal amplitude following the first injection of ATP: ~ 20% (*n* = 4; results from **Fig. 3B** and **S4A-S4C**).


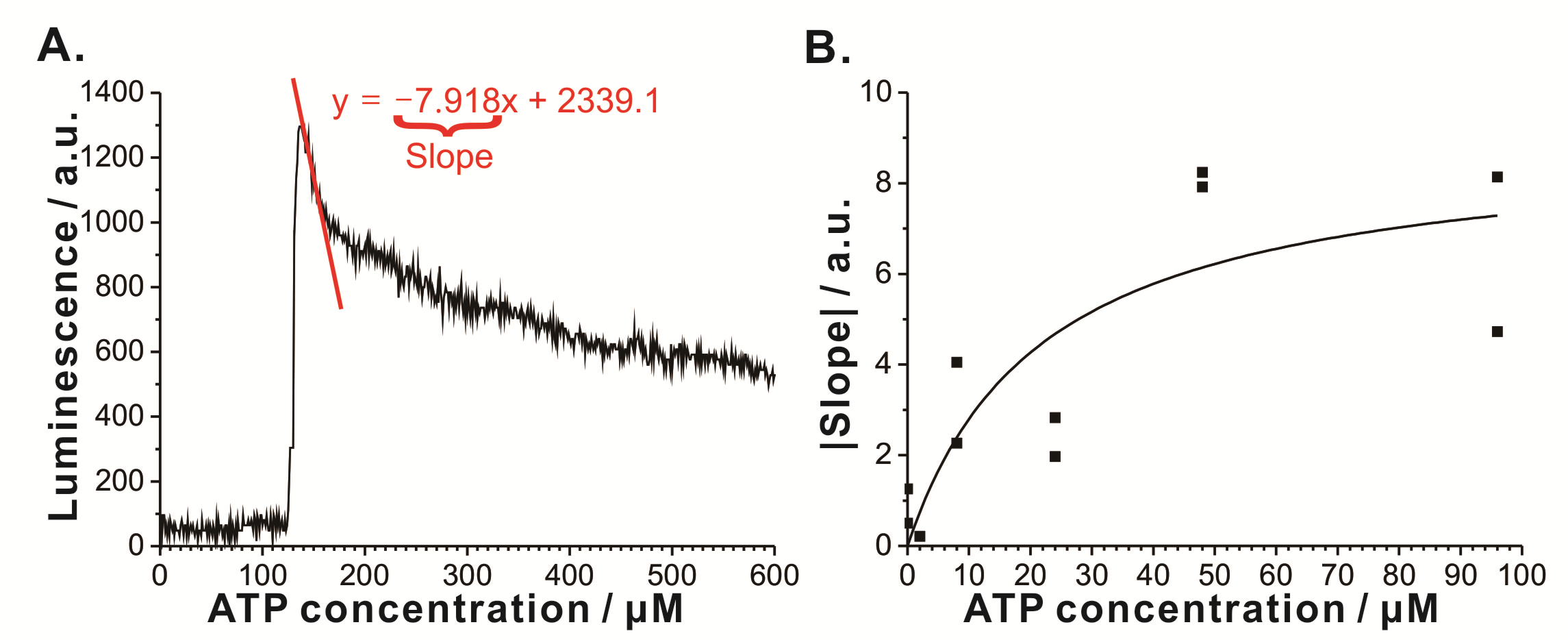


**Figure S5.** Chemiluminescence decay as a proxy for reaction rate. (A) 48 μM ATP measured using the proposed system using luciferin/luciferase reaction. The red line was fitted to the data points recorded during a 50-s period starting from the time point when chemiluminescence maximum was observed. The slope value was extracted from the equation describing this line. (B) A non-linear function *Slope* = (8.95 × *C*)/(22.0 + *C*) fitted to the data series relating the chemiluminescence decay slope with concentration. The apparent Michaelis-Menten constant was estimated to be ~ 22 μM ATP. The mean squared error of the fitted model in (B) is ~ 3.07 a.u.


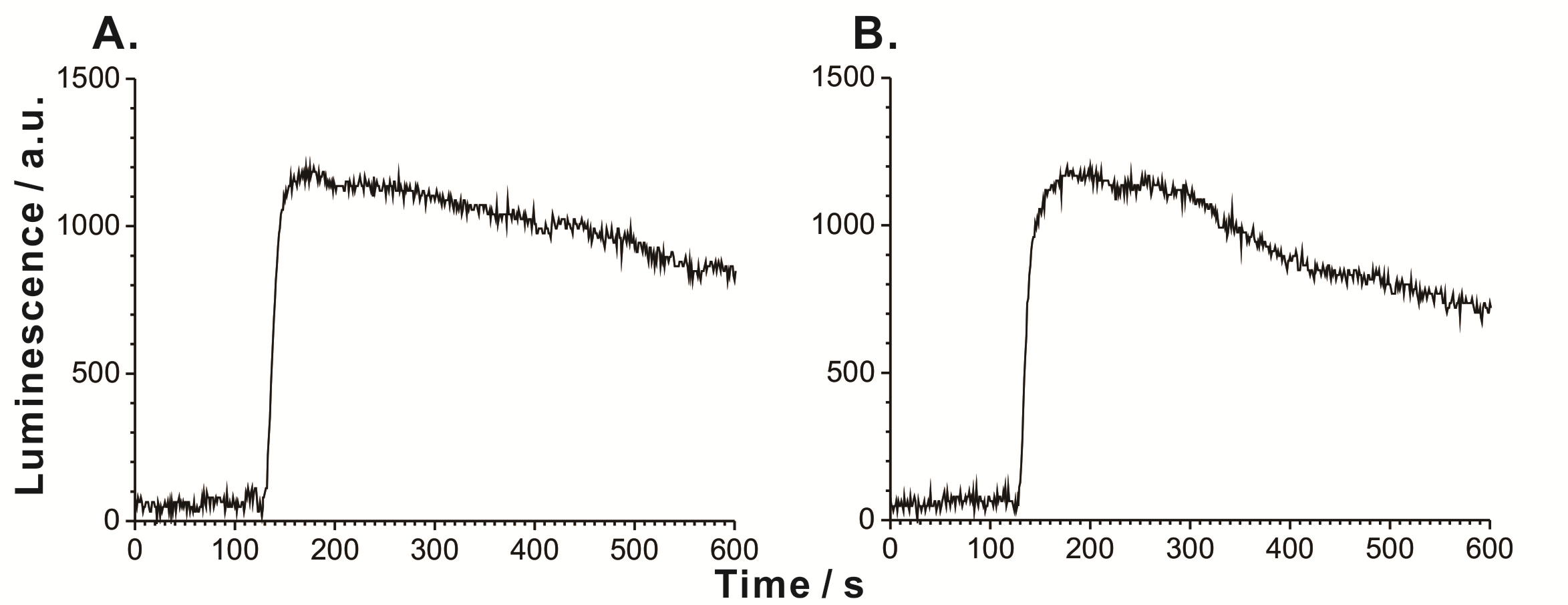


**Figure S6.** Verification of the influence of AMP on the luciferin/luciferase reaction. An aliquot of ATP (16 μL 1 mM stock solution) was injected at the beginning of experiment (120 s after the start of data acquisition) to the final concentration 32 μM. (A) Control – no AMP added to the reaction mixture. (B) 0.2 mM AMP present in the reaction mixture. In this experiment (A and B), ATP compensation was disabled.


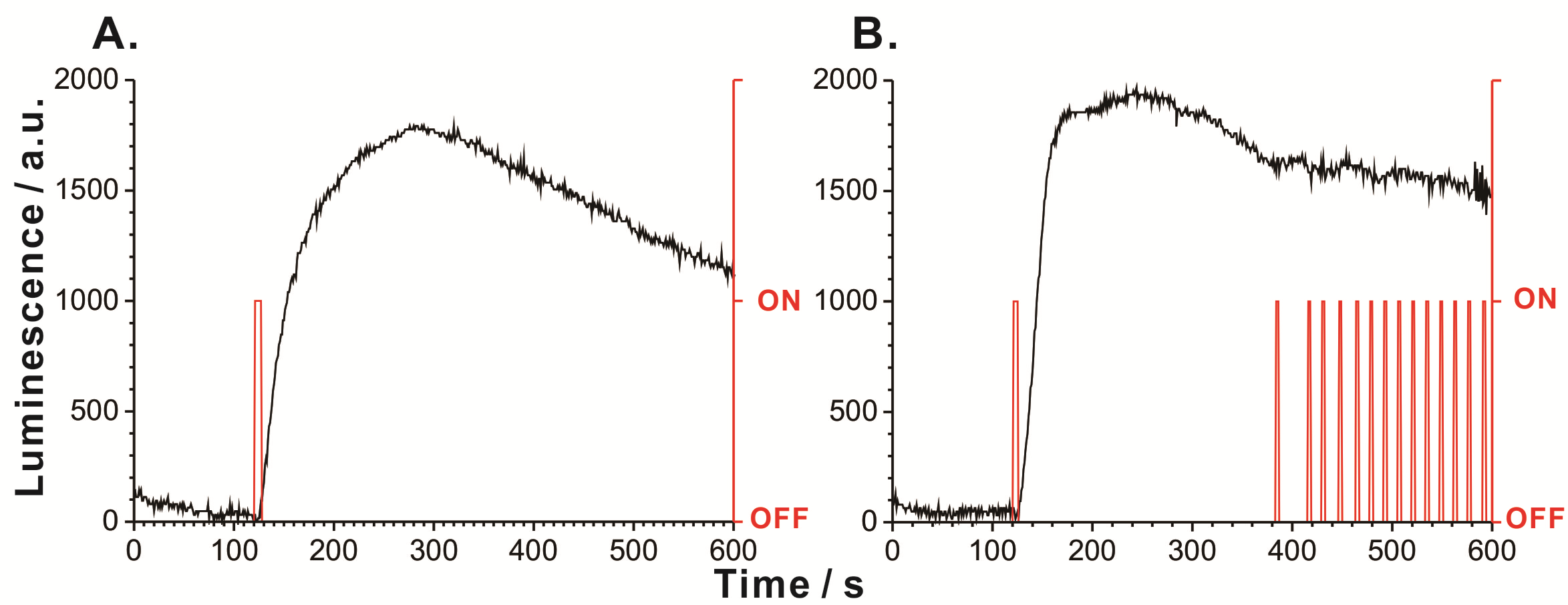


**Figure S7.** Verification of the possibility to use the bio-opto-electronic system for the compensation of ATP while using ATP concentrations higher than the linear range of the chemiluminescence detection shown in **Fig. S2**. ATP concentration following first injection: 0.2 mM (10 μL 10 mM ATP solution). Subsequent (compensatory) injections: 5 μL 10 mM ATP. Apyrase activity: 20 mU. (A) ATP compensation disabled. (B) ATP compensation enabled.


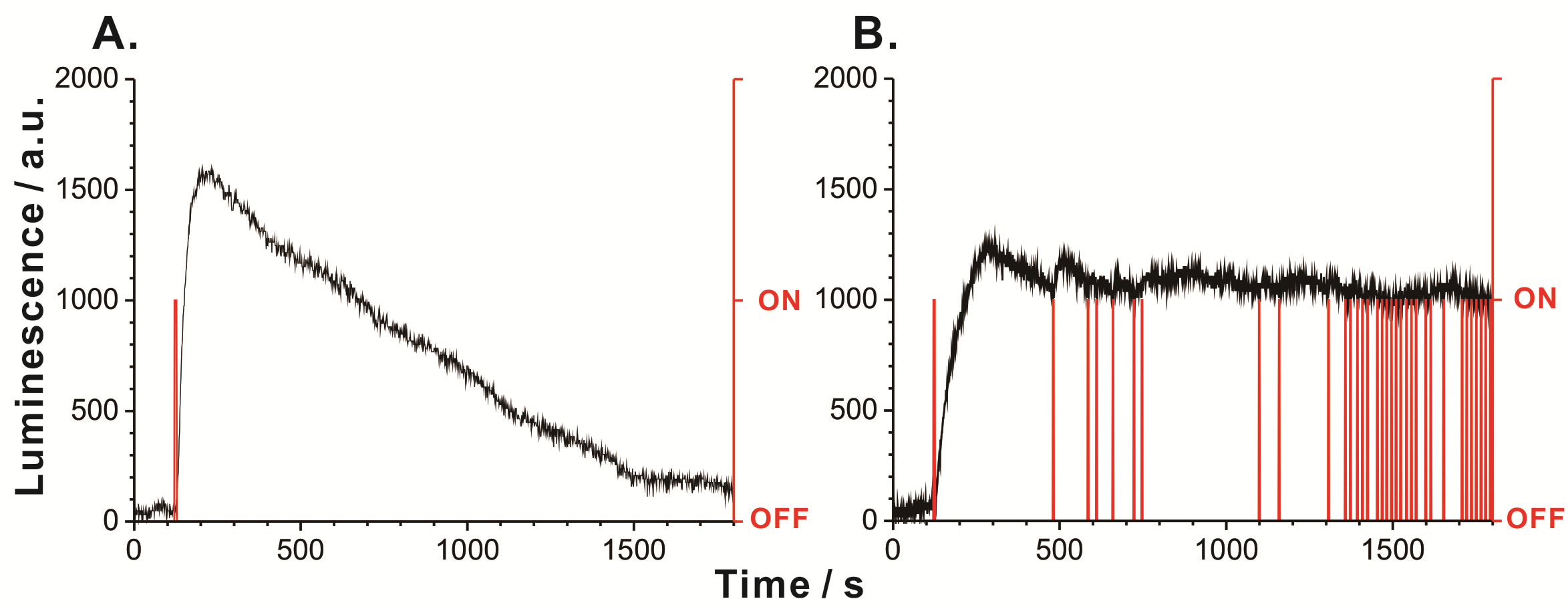


**Figure S8.** Verification of the influence of a real sample on the operation of the bio-opto-electronic system for compensation of ATP. A 100 μL aliquot of Hs 578T cell extract (prepared according to the procedure described above) was added to the reaction mixture (final volume: 500 μL). An aliquot of ATP (16 μL 1 mM stock solution) was injected at the beginning of experiment (120 s after the start of data acquisition) to the final concentration 32 μM. (A) ATP compensation disabled. (B) ATP compensation enabled.
